# Identification of Three Type II Toxin-Antitoxin Systems in *Streptococcus suis* Serotype 2

**DOI:** 10.3390/toxins10110467

**Published:** 2018-11-13

**Authors:** Jiali Xu, Nian Zhang, Manman Cao, Sujing Ren, Ting Zeng, Minglu Qin, Xigong Zhao, Fangyan Yuan, Huanchun Chen, Weicheng Bei

**Affiliations:** 1 State Key Laboratory of Agricultural Microbiology, The Cooperative Innovation Center for Sustainable Pig Production, College of Veterinary Medicine, Huazhong Agricultural University, Wuhan 430070, China; jialixu@webmail.hzau.edu.cn (J.X.); nianzhang@webmail.hzau.edu.cn (N.Z.); mmcao@webmail.hzau.edu.cn (M.C.); rensujing0327@163.com (S.R.); tingzeng@webmail.hzau.edu.cn (T.Z.); mlqin199591@126.com (M.Q.); xgzhao@webmail.hzau.edu.cn (X.Z.); chenhch@mail.hzau.edu.cn (H.C.); 2 Key Laboratory of Prevention and Control Agents for Animal Bacteriosis, Institute of Animal Husbandry and Veterinary Sciences, Hubei Academy of Agricultural Sciences, Wuhan 430064, China; fangyanyuan12@163.com

**Keywords:** RelBE, ParDE, cell filamentation, autoregulation

## Abstract

Type II toxin-antitoxin (TA) systems are highly prevalent in bacterial genomes and have been extensively studied. These modules involve in the formation of persistence cells, the biofilm formation, and stress resistance, which might play key roles in pathogen virulence. SezAT and *yefM-yoeB* TA modules in *Streptococcus suis* serotype 2 (*S. suis* 2) have been studied, although the other TA systems have not been identified. In this study, we investigated nine putative type II TA systems in the genome of *S. suis* 2 strain SC84 by bioinformatics analysis and identified three of them (two *relBE* loci and one *parDE* locus) that function as typical type II TA systems. Interestingly, we found that the introduction of the two RelBE TA systems into *Escherichia coli* or the induction of the ParE toxin led to cell filamentation. Promoter activity assays indicated that RelB1, RelB2, ParD, and ParDE negatively autoregulated the transcriptions of their respective TA operons, while RelBE2 positively autoregulated its TA operon transcription. Collectively, we identified three TA systems in *S. suis* 2, and our findings have laid an important foundation for further functional studies on these TA systems.

## 1. Introduction

*Streptococcus suis* (*S. suis*) is an important major swine and zoonotic pathogen that causes severe infection [1,2,3]. It is associated with a variety of serious diseases, including arthritis, septicemia, pneumonia, endocarditis, and meningitis in pigs and leads to great economic losses worldwide [4,5]. *S. suis* infection in humans causes arthritis, septicemia, meningitis and streptococcal toxic shock syndrome (STSS) through direct contact with sick pigs or pork by-products [6,7]. According to the composition of capsular polysaccharide (CPS), 33 serotypes (types 1 to 31, 33, and 1/2) in *S. suis* have been described [8]. Among them, serotype 2 is acknowledged as the most prevalent and virulent serotype, and it has been frequently isolated from diseased pigs [2]. Two large outbreaks of human *S. suis* 2 infection occurred in 1998 and 2005 in China, causing great public concern due to the high pathogenicity of this zoonotic pathogen in humans [9]. However, the pathogenesis of *S. suis* 2 infection has not been well understood yet. Previously, we have predicted 9 type II Toxin-antitoxin (TA) systems in the genome of *S. suis* SC84 by bioinformatics analysis [10]. The *yefM-yoeB* TA system in *S. suis* 2 has been described, but this does not contribute to virulence in the murine infection model [10]. The SezAT module has firstly been characterized as an active TA system in *S. suis* 2 strain 05ZYH33, which promotes the maintenance of the SsPI-1 pathogenicity island [11], yet its function remains unclear. First and foremost, other TA systems, prevalent in *S. suis* 2, should be identified, and their functions should be explored. This would provide further insight into the mechanisms of severe *S. suis* 2 infection.

Recently, type II TA systems, in which toxins and antitoxins are proteins, have been widely prevalent in bacterial genomes and the most extensively studied [12]. Based on the sequence similarity and nature of toxins, several super-families of type II TA modules have been defined [13,14]. Multiple studies have reported some super-families of type II TA systems, including MazEF, RelBE, HigBA, HipBA, VapBC, Phd/Doc, CcdAB, HicAB and ParDE [15,16,17,18,19]. MazEF in *Escherichia coli* [18,20], *Mycobacterium tuberculosis* [21], S*taphylococcus equorum* [22] and *Streptococcus mutans* [23] has been well characterized. The role of RelBE, as one of the most common type II systems, in important physiological processes of *Vibrio cholerae* has been described [14]. Seventeen TA pairs have been identified in *V. cholera*, of which, seven RelBE, three ParDE, two HigBA, and a single Phd/Doc were well characterized [14,24,25,26]. The *hipBA* locus in *M*. *tuberculosis*, as a ubiquitous TA locus, has been found to be involved in stress response [27]. MazEF, VapBC and Phd/Doc, the three putative TA modules of *Mycobacterium smegmatis*, play an essential role in cell survival [28]. Moreover, type II TA systems have been found to contribute to the virulence of *Salmonella* [29] and *Leptospira interrogans* [30]. There is increasing evidence that type II TA systems are ubiquitous and strongly related to the formation of persistence cells, the regulation of biofilm formation, stress resistance, and other biological processes, and that they contribute to bacterial pathogenicity [13,15,31,32]. Thus, the identification of novel type II TA systems in pathogenic microbes, especially *S. suis* 2, is urgently needed.

This study identified three active type II TA systems in *S. suis* 2, including two RelBE and one ParDE. A total of five TA systems, present in *S. suis* 2, have been found and described for the first time. Our results have provided new information for further functional studies on these TA systems.

## 2. Results

### 2.1. Identification of Putative Type II TA Systems in S. suis 2

The putative type II TA loci in *S. suis* SC84 were predicted with TAfinder, a newly developed online tool in TADB (Toxin-Antitoxin Database, http://202.120.12.135/TADB2/Introduction.html#table_s2), which can quickly detect the TA prediction [12]. Nine putative type II TA systems are shown in Table 1 and distributed widely in the circular complete genome map (Figure S1). Both the nine putative TA systems and the ID numbers of each putative toxin or antitoxin are included in the Figure S1. The genetic organization of putative type II TA systems are shown in Figure S2. Each antitoxin gene is located upstream of the toxin gene, except for the putative TA_2, which is associated with the direction of the gene encoding (Figure S2). It is interesting that TA_2 and TA_3 were found to share the same toxin gene, according to the predicted result, which may need to be further researched. Additionally, the physical distance (in bp) between the putative antitoxin and toxin coding sequences, TA protein domain pair, and TA family (based on the toxin protein) are shown in Table 1. While the TA_4 system was found to be entirely homologous to SezAT in *S. suis* 05ZYH33 [11], suggesting that TA_4 works as typical type II TA systems, TA_8 was identified as the *yefM-yoeB* system in *S. suis* SC84 [10]. Therefore, the remaining seven modules, including five modules belonging to the *relBE*/*parDE* family, were regarded as putative type II TA systems and chosen for further study.

### 2.2. Each Putative Type II TA Locus Was Encoded by An Operon

**Figure 1:**
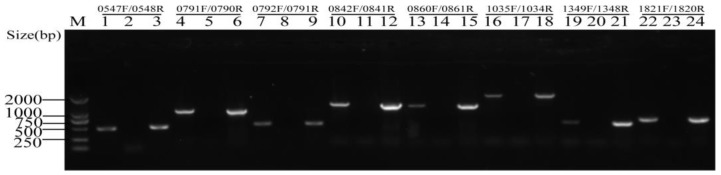
Co-transcription analysis of putative type II toxin-antitoxin (TA) modules in *S. suis* 2. The total RNA was isolated from the logarithmic phase *S. suis* 2 and used to synthesize cDNAs. PCR was carried out, with primer pairs indicated above the lanes. Lanes 1, 4, 7, 10, 13, 16, 19 and 22 represent the amplification using cDNAs as the template; Lanes 2, 5, 8, 11, 14, 17, 20 and 23 represent the amplification using cDNA- (RNA converted into a cDNA reaction without reverse transcriptase) as the template; and lanes 3, 6, 9, 12, 15, 18, 21 and 24 represent the amplification using genomic DNA (gDNA) as the template. Lane M indicates the DL2000 DNA Marker.

The two genes of the TA module were organized into an operon, and the TA module was verified by RT-PCR analysis. Therefore, the total RNA was extracted from *S. suis* 2 and used to synthesize cDNAs. Then, cDNAs were PCR amplified using the assigned primer pairs, which anneals to the 5′-end and 3′-region of the coding sequence of each putative TA locus. The result of RT-PCR analysis (Figure 1) showed that the expected sizes of PCR products were in line with those of the genomic DNA (gDNA). No PCR products were detected in the negative controls (cDNA-), in which the reverse transcription operated without the reverse transcriptase, therefore eliminating genomic DNA contamination. These data indicated that, for each putative TA module, the toxin genes and antitoxin genes were actively co-transcribed and organized into a bicistronic operon.

### 2.3. Effects of Each Putative TA System on the Growth of E. coli Using the Selective Expression Vector pETBAD

**Figure 2:**
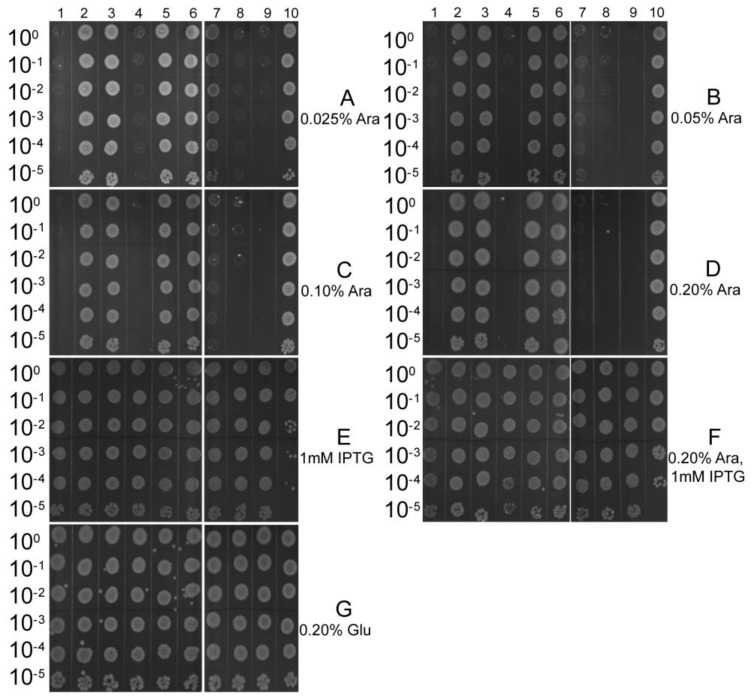
Effect of the induction of nine putative toxin-antitoxin (TA) modules, cloned separately into the selective expression vector, on *E. coli* growth. *E. coli* BL21 (DE3) pLysS cells, harboring the constructed pETBAD-0547-0548 (Lane 1), -0790-0791 (Lane 2), -0792-0791 (Lane 3), -0842-0841 (Lane 4), -0860-0861 (Lane 5), -1035-1034 (Lane 6), -1349-1348 (Lane 7), -1818-1817 (Lane 8), -1821-1820 (Lane 9), and pETBAD (Lane 10), were grown to OD_600_ of 0.6–0.8. Each culture was serially diluted, and 5-µL drops, with the concentration ranging from 10^0^ (top) to 10^−5^ (bottom), were respectively spotted onto the plate, with different inducers. (**A**–**D**) 0.025%, 0.05%, 0.10%, and 0.20% l-arabinose were added successively to induce expression of putative toxins; (**E**) 1 mM isopropyl β-d-thiogalactopyranoside (IPTG) was added to induce the expression of putative antitoxins; (**F**) Both 0.20% l-arabinose and 1 mM IPTG were added to induce the expression of putative TA modules; (**G**) 0.20% d-glucose (repression conditions) was added as the control.

Primarily, we chose the selective expression vector, pETBAD [10], to determine whether the putative TA systems could typically be described as the phenomenon of toxins that are toxic to *E. coli* being susceptible of neutralization by their cognate antitoxins. The plasmid of pETBAD was generated in order to independently control the expression of toxins by arabinose or the expression of antitoxins by isopropyl β-d-thiogalactopyranoside (IPTG). The plasmids of the pETBAD-antitoxin-toxin (pETBAD-0547-0548, -0790-0791, -0792-0791, -0842-0841, -0860-0861, -1035-1034, -1349-1348, -1818-1817 and -1821-1820) and pETBAD were transformed into *E. coli* BL21 (DE3) pLysS cells, respectively. BL21 (DE3) pLysS cells, harboring the constructed plasmids of pETBAD-0547-0548, -0842-0841, -1349-1348, -1818-1817, and -1821-1820, exhibited a significant growth inhibition, compared to those containing pETBAD, with increasing concentrations of l-arabinose. BL21 (DE3) pLysS cells, harboring the constructed plasmids of pETBAD-0790-0791, -0792-0791, -0860-0861, and -1035-1034, exhibited almost identical growth, compared to those containing the pETBAD plasmid, under the same conditions (Figure 2A–D). However, no major difference in *E. coli* growth was observed between the co-induction of BL21 (DE3) pLysS cells, harboring the constructed plasmids of the pETBAD-antitoxin-toxin (pETBAD-0547-0548, -0790-0791, -0792-0791, -0842-0841, -0860-0861, -1035-1034, -1349-1348, -1818-1817 and -1821-1820) and pETBAD (Figure 2F), and the induction of each putative antitoxin alone (Figure 2E), which was almost consistent with the *E. coli* growth of the control group (Figure 2G). Our results indicated that the loci, TA_1, TA_4, TA_7, TA_8 and TA_9, potentially work as typical type II TA systems. As TA_4 and TA_8 have been studied previously [10,11], we carried out the following experiments to further confirm the toxic effects of other TA systems.

### 2.4. Evaluation of the Toxic Effects of Putative Toxins on the Growth of E. coli

**Figure 3:**
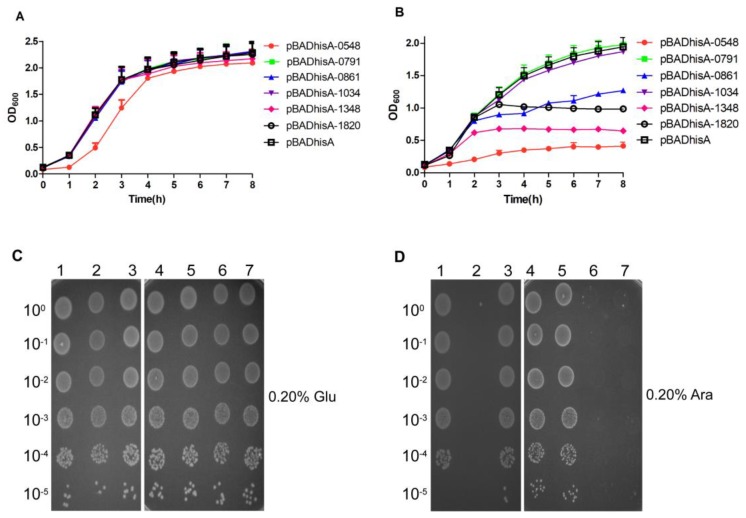
Effect of the induction of putative toxins on the growth of *E. coli*. *E. coli* Top10 cells, harboring the corresponding plasmids, were grown to OD_600_ of 0.05–0.20. Each culture was then divided into two equal volumes and supplemented with either 0.20% d-glucose (**A**) or 0.20% l-arabinose (**B**). Culture growth was monitored by measuring OD_600_ every hour. Growth curves are representative of at least 3 independent experiments. Top10 cells, harboring the corresponding pBADhisA-0548 (Lane 2), -0791 (Lane 3), -0861 (Lane 4), -1034 (Lane 5), -1348 (Lane 6), -1820 (Lane 7), and pBADhisA (Lane 1), were grown to OD_600_ of 0.6–0.8. Each culture was serially diluted, and 5-µL drops, with the concentration ranging from of 10^0^ (top) to 10^−5^ (bottom), were successively spotted onto the different plates with (**C**) 0.20% d-glucose or (**D**) 0.20% l-arabinose.

The pBADhisA plasmid was used to determine whether the putative toxins were toxic to *E. coli*, and we cloned the putative toxin genes into the pBADhisA expression vector. TA_2 and TA_3 had the same toxin gene, SSUSC84_0791. Therefore, the six plasmids of the pBADhisA-toxin (pBADhisA-0548, -0791, -0861, -1034, -1348, and -1820) and pBADhisA were transformed into *E. coli* Top10 cells. In the presence of 0.20% d-glucose (repression conditions), Top10 cells, harboring the plasmid of pBADhisA-0548 demonstrated a moderate growth defect, compared to those harboring the plasmid of pBADhisA, while Top10 cells, harboring the plasmids of pBADhisA-0791, -0861, -1034, -1348, and -1820, exhibited no major difference in *E. coli* growth (Figure 3A). Under induction conditions, Top10 cells, containing pBADhisA-0791 and pBADhisA-1034, exhibited no growth inhibition, compared to those containing pBADhisA. Under the same induction conditions, Top10 cells, carrying pBADhisA-0548, -0861, -1348, and -1820, exhibited growth inhibition, unlike those carrying pBADhisA (Figure 3B).

Overnight cultures of *E. coli* Top10 cells, carrying the plasmids of pBADhisA-0548, -0791, -0861, -1034, -1348, -1820 or pBADhisA (control), were diluted at 1:100 in LB-ampicillin, grown to OD_600_ of 0.6–0.8 and serially diluted, and 5-µL drops were spotted onto the different plates with 0.20% d-glucose or 0.20% l-arabinose. As shown in Figure 3C,D, the Top10 cells, carrying pBADhisA-0548, -1348 and -1820 (toxin genes of TA_1, TA_7, and TA_9) on the plate with 0.20% l-arabinose, exhibited obvious growth inhibition, while the Top10 cells, carrying pBADhisA-0791, -0861, and -1034, exhibited normal growth (Figure 3D). By contrast, no major difference in growth, between Top10 cells, carrying pBADhisA-0548, -0791, -0861, -1034, -1348, and -1820, and those carrying pBADhisA on the plate with 0.20% d-glucose was observed (Figure 3C). These results indicated that TA_1, TA_7, and TA_9 are potential type II TA systems.

### 2.5. Evaluation of the Antitoxic Effects of Putative Antitoxins

**Figure 4:**
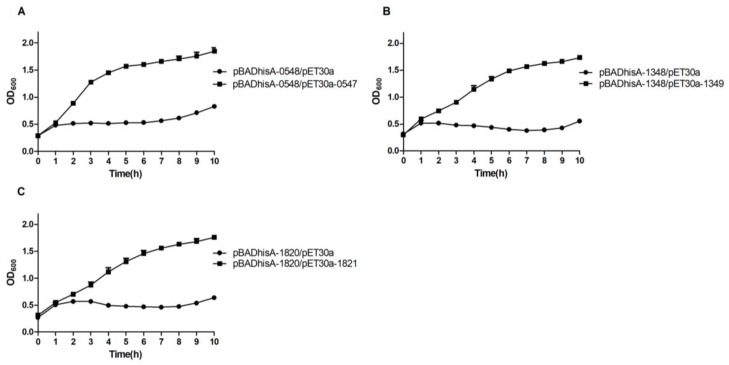
Effect of the co-expression of each putative antitoxin and its cognate toxin on the growth of *E. coli*. BL21 (DE3) pLysS cells, harboring the constructed (**A**) pBADhisA-0548 and pET30a-0547 (or pET30a, as control); (**B**) pBADhisA-1348 and pET30a-1349 (or pET30a, as control); and (**C**) pBADhisA-1820 and pET30a-1821 (or pET30a, as control), which were grown to OD_600_ of 0.2–0.3. Each culture was supplemented with both 0.20% l-arabinose and 1.00 mM isopropyl β-d-thiogalactopyranoside (IPTG). Culture growth was evaluated by measuring OD_600_ every hour. Growth curves are representative of at least 3 independent experiments.

To determine whether the toxic effect of each toxin can be alleviated by its cognate antitoxin, the sequence of each putative antitoxin was cloned separately into the pET30a plasmid. Additionally, the resulting plasmids were designated as pET30a-antitoxin (pET30a-0547, -1349, and -1821). *E. coli* BL21 (DE3) pLysS cells, harboring pBADhisA-0548 and pET30a-0547 (or pET30a, as control), pBADhisA-1348 and pET30a-1349 (or pET30a, as control), and pBADhisA-1820 and pET30a-1821 (or pET30a, as control), were successfully obtained by co-transformation and selection with ampicillin and kanamycin. When BL21 (DE3) pLysS cells, harboring these plasmids, were grown to OD_600_ of 0.20–0.30, each culture was supplemented with 0.20% l-arabinose and 1 mM IPTG. The induction of TA_1 (Figure 4A), TA_7 (Figure 4B), and TA_9 (Figure 4C) resulted in normal growth, while BL21 (DE3) pLysS cells, carrying both pBADhisA-toxin and pET30a plasmids, demonstrated a significant growth inhibition (Figure 4A–C), suggesting that the putative antitoxins of TA_1, TA_7, and TA_9 could neutralize the cognate toxin.

Taken together, these above results indicated that TA_1, TA_7, and TA_9 loci work as typical type II TA systems.

**Figure 5:**
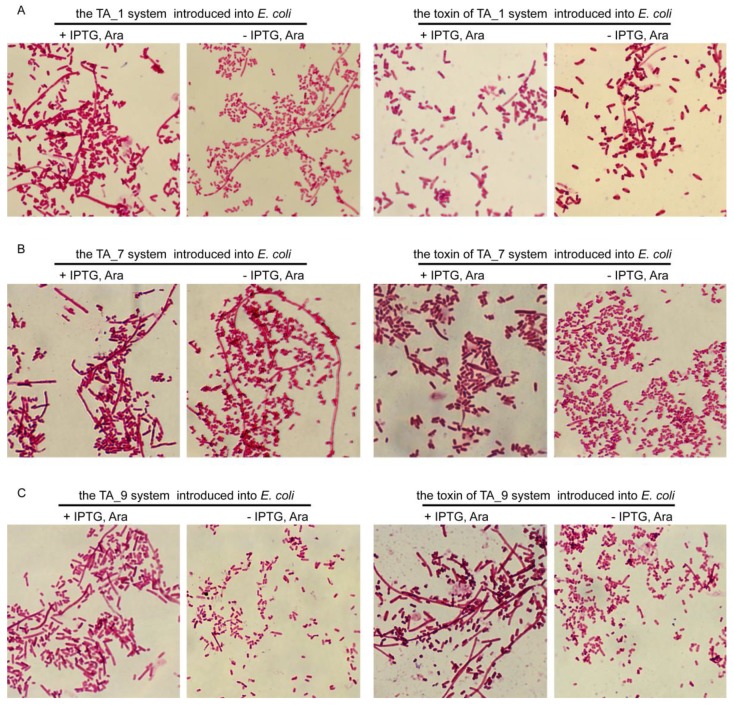
Introduction of the *S. suis* toxin or toxin-antitoxin (TA) complex into *E. coli* leads to cell filament formation. (**A**) The TA_1 system or the toxin of the TA_1 system, (**B**) the TA_7 system or the toxin of the TA_7 system, and (**C**) the TA_9 system or the toxin of the TA_9 system were introduced into *E. coli* BL21 (DE3) pLysS cells and were induced with 1 mM isopropyl β-d-thiogalactopyranoside (IPTG) and 0.20% l-arabinose (+IPTG, Ara) and without IPTG and l-arabinose (−IPTG, Ara) (as control). Light microscope morphology of *E. coli* cells, using Gram staining (×100), was performed at 5 h after induction.

During these growth experiments, microscopic examination showed that normal-sized cells become filamentous after the induction of TA_1, TA_7, and TA_9 systems (Figure 5A–C). The TA_1 and TA_7 systems were introduced into *E. coli* and caused cell filamentation, with or without the inducers (IPTG, l-arabinose) at 5 h. The induction of the whole TA_9 system or the toxin of TA_9 alone in *E. coli* exhibited cell filamentation, while no cell filamentation was observed in the absence of inducers (−IPTG, l-arabinose) (Figure 5C). Therefore, the introduction of TA_1 and TA_7 systems into *E. coli* led to cell filamentation. Additionally, the induction of the toxin of TA_9 caused more significant cell filamentation, compared that of the whole TA_9 system. It was reported that the cell morphology, induced by the toxin, ParE, was filamentous in *E. coli* [33] and *Caulobacter crescentus* [34]. After confirming that TA_1, TA_7, and TA_9 loci work as typical type II TA systems, the toxin of TA_1 was renamed RelE1, the toxin of TA_7 was renamed RelE2, and the toxin of TA_9 was renamed ParE. The antitoxins of TA_1, TA_7, and TA_9 were respectively renamed RelB1, RelB2, and ParD.

### 2.6. Antitoxin or TA Complex Autoregulates the TA Operon

**Figure 6:**
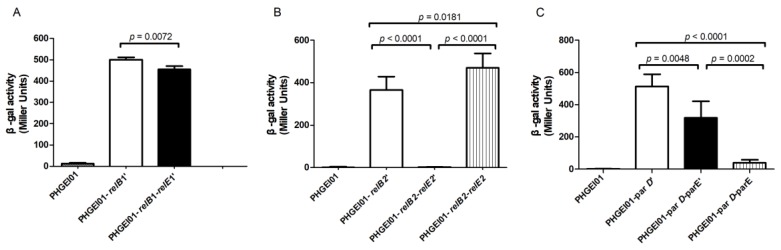
The antitoxin or TA complex autoregulates the TA operon. (**A**) The RelBE1 system; (**B**) The RelBE2 system; (**C**) The ParDE system. *E. coli* WM3064 cells, harboring the corresponding reporter plasmids, were collected in the mid-exponential phase (OD_600_~0.6–0.7) and tested for β-galactosidase activity. The descriptive data of the means ± standard deviations for three of the independent experiments are shown. The statistical significance was tested using a one-tailed unpaired *t* test. A *p* < 0.05 was considered statistically significant.

In typical type II TA systems, the antitoxin alone or the TA complex binds to the promoter and regulates the transcription of the TA operon [35]. The β-galactosidase activity was measured in order to study the autoregulation of the three TA (relBE1, relBE2, and parDE) operons, as previously described [36,37]. The plasmids of pHGEI01-antitoxin’ (-relB1’, -relB2’, and -parD’), pHGEI01-antitoxin-toxin’ (-relB1-relE1’, -relB2-relE2’, and -parD-parE’), and pHGEI01-antitoxin-toxin (relB1-relE1, -relB2-relE2, and -parD-parE) were constructed and transformed into *E. coli* WM3064, respectively. As for the RelBE1 system, the promoter activity in WM3064 cells, carrying the pHGEI01-relB1’ plasmid, was significantly higher than that in cells carrying pHGEI01-relB1-relBE1’ (*p* = 0.0072) (Figure 6A), indicating that the antitoxin, RelB1, repressed the promoter activity. However, it is a pity that we failed to construct the pHGEI01-relB1-relE1 after several attempts. As for the RelBE2 system, the promoter activity was obviously repressed by the antitoxin, RelB2, but it was enhanced by the RelBE2 TA complex (Figure 6B). Concerning the ParDE system, it was found that the promoter activity in WM3064 cells, carrying the pHGEI01-parD’ plasmid, was significantly higher than that in cells carrying pHGEI01-parD-parE’ (*p* = 0.0048) and pHGEI01-parD-parE (*p* < 0.0001). Moreover, it was found that the promoter activity in WM3064 cells, carrying the pHGEI01-parD-parE’ plasmid, was significantly higher than that in cells carrying pHGEI01-parD-parE (*p* = 0.0002) (Figure 6C), indicating that ParD and ParDE repressed the promoter activity. Additionally, it was confirmed that the ParDE complex repressed the promoter activity more significantly than ParD. Furthermore, concerning the RelBE2 system, the inhibitory effect of antitoxin, RelB2, on the transcription of the TA operon was reversed by its cognate toxin, RelE2. As for the ParDE system, the toxin ParE helped the antitoxin, ParD, to repress the promoter activity. Taken together, these results indicated that RelB1, RelB2, ParD, and ParDE negatively autoregulated the transcriptions of their respective TA operons, while RelBE2 positively autoregulated its TA operon transcription.

## 3. Discussion

The type II TA modules are ubiquitous in various bacteria and archaea [12,29,38,39] and have drawn worldwide attention in recent years. For example, the VapBC TA systems in *M. tuberculosis* [40,41] and *Streptomyces sp*. [36] were described. RelBE, belonging to a new TA family, was reported in *E. coli* K-12 [42]. The *yefM-yoeB* locus in *S. suis* [10], *Streptococcus pneumoniae* [43], and *Staphylococcus aureus* [44,45] was described. SezAT in *S. suis* [11] and PezAT in *S*. *pneumoniae* [46,47,48] were described. Many type II TA systems in various important pathogens, especially *M. tuberculosis* [27,40,41,49] and *S*. *pneumoniae* [43,46,47,48,50,51,52], which could lead to severe infection in humans, have been well studied.

In this study, we chose the TAfinder tool and found nine putative TA systems in *S. suis* SC84. We successfully constructed the related plasmids (pETBAD-antitoxin-toxin, pBADhisA-toxin, and pET30a-antitoxin) and identified three TA systems (two RelBE and one ParDE), which have previously been uncharacterized in *S. suis* SC84. The expression of SSUSC84_0791, SSUSC84_0861, and SSUSC84_1034 exhibited no major growth defect (Figure 2A–D and Figure 4B), suggesting that TA_2, TA_3, TA_5, and TA_6 loci may not work as active TA systems. However, the effect of SSUSC84_0861 induction on *E. coli* growth in the liquid medium (Figure 3B) was found to be inconsistent with that on the solid medium (Figure 2A–D and Figure 3D). It was not observed that the product of the gene, SSUSC84_0861, could inhibit the growth of *E. coli* on the solid medium (Figure 2A–D and Figure 3D). Therefore, it remains to be explored whether or not SSUSC84_0861 works as a toxin in the future. Therefore, the effect of the plasmid of the pETBAD-antitoxin-toxin alone on *E. coli* growth is equal to the effect of both the pBADhisA-toxin and pET30a-antitoxin, under the condition of the induction of the toxin and its cognate antitoxin in vitro. It is convenient to use the selective expression vector, pETBAD, to demonstrate the effect of toxin-antitoxin systems on *E. coli* growth [10].

Structural analysis indicates that the toxin, ParE, is homologous to RelE and YoeB toxins, but the cellular target of ParE is different from that of RelE [34]. The RelE protein cleaves mRNA in the ribosomal A-site to inhibit translation [53,54], while ParE has been distinguished by inhibiting DNA gyrase and thereby blocking DNA replication [33]. The *yefM-yoeB* has been fused with the *relEB* family, while YoeB exhibits a similar tertiary fold to RelE [55]. As the previous report found that the toxin, StbE_pEP36_, did not induce cell filamentation [56], and we also found the toxins RelE1 and RelE2 did not induce cell filamentation (Figure 5A,B). The mechanism by which the introduction of the two RelBE systems into *E. coli* leads to cell filamentation (Figure 5A,B), and the introduction of *yefM-yoeB* inhibits *E. coli* growth, under both the repression and induction conditions [10], remains to be explored in the future. Additionally, the induction of the toxin, ParE, led to cell filamentation in *S*. *pneumoniae* [51], while the induction of the ParD/ParE complex or ParE in *E. coli* exhibited different lengths of cells (Figure 5C), which may be consistent with the finding that the ParE toxin can inhibit cell division and that ParD prevents the inhibition by blocking the binding of ParE to gyrase and reducing cell damage [33]. The result that the ParD protein repressed the expression of the *parDE* operon of *S. suis* 2 is consistent with the function of ParD, as a transcriptional repressor in *E. coli* [57]. We also found that the ParDE complex repressed the promoter activity more significantly than ParD in *S. suis* 2 (Figure 6C), suggesting that the toxin, ParE, helped the antitoxin, ParD, to repress the promoter activity. However, the MParD2 repressed the promoter activity more strongly than MParDE2 (encoded by the *parDE*2 gene of *M. tuberculosis*) in the surrogate *M. smegmatis* [58]. These results indicated that ParD and ParDE negatively autoregulated the transcriptions of their respective TA operons. The ParDE TA system in other bacteria was well characterized [24,33,57,58,59], which might help to elucidate the functions of ParDE in *S. suis* 2 in further studies.

In *E. coli*, RelE was confirmed to enhance the repressor activity of RelB [53,60]. Additionally, the RelBE2sca complex or RelB2sca was found to repress the transcription of the TA operon in *Streptomyces cattleya* DSM46488 [61]. We failed to construct the pHGEI01-*relB*1-*relE*1 plasmid due to several base mutations. Therefore, it remains unclear how the RelBE1 complex regulates the transcription of the TA operon. There is a new finding that RelJ, RelBE, RelFG, and RelJK function as transcriptional repressors, and RelB and RelF function as transcriptional activators, by β-galactosidase activity analysis [49]. This study found, first, that the RelBE2 complex positively regulates the promoter activity (Figure 6B), while the RelBE of other bacteria negatively regulates the promoter activity [49,53,60,61]. Considering that type II TA systems contribute to the formation of persistence cells, stress response, biofilm formation and other various biological processes [13,15,31,62,63,64], the mechanism of three TA systems in *S. suis* 2 remains to be further explored in future studies.

In summary, we have identified two RelBE TA systems and one ParDE TA system in *S. suis* SC84. We also found that the introduction of the two RelBE TA systems into *E. coli* or the induction of the ParE toxin led to cell filamentation and that RelB1, RelB2, ParD, and ParDE negatively autoregulated the transcriptions of their respective TA operons, while RelBE2 positively autoregulated its TA operon transcription by promoter activity assays. However, the functions of three TA systems in *S. suis* 2 need to be intensively studied.

## 4. Materials and Methods

### 4.1. Bacterial Strains, Plasmids, Primers, and Growth Conditions

Bacterial strains and plasmids used in this study are listed in Table S1. Primers are listed in Table S2. The *S. suis* 2 strain was cultured in Tryptic Soy Broth (TSB) or on Tryptic Soy Agar (TSA; Difco Laboratories, Detroit, MI, USA), supplemented with 10% (*v*/*v*) newborn bovine serum at 37 °C. *E. coli* strains were cultured in Luria-Bertani (LB) broth or on LB agar at 37 °C. When required, antibiotics were added at the following concentrations: 75 μg/mL ampicillin or 25 μg/mL kanamycin for *E. coli*.

### 4.2. Bioinformatics Analysis, RNA Isolation and RT-PCR Analysis

The putative type II TA systems in the *S. suis* 2 strain SC84 were predicted with TAfinder (http://202.120.12.133/TAfinder/TAfinder.php). In our work, *S. suis* 2 was grown to the mid-log-phase and was used to extract the total RNA. The total RNA was purified by using an SV (spin or vacuum) total RNA isolation system (Promega, Madison, WI, USA), according to the manufacturer’s protocol. The RNA integrity and concentrations were determined by agarose gel electrophoresis and NanoDrop, respectively. The cDNAs were generated from these RNA samples with HiScript II Q RT SuperMix (Vazyme, Nanjing, China). We used the specific primers (0547F/0548R, 0791F/0790R, 0792F/0791R, 0842F/0841R, 0860F/0861R, 1035F/1034R, 1349F/1348R, and 1821F/1820R) in Table S2 to confirm the co-transcription of putative toxin genes and antitoxin genes.

### 4.3. Putative TA Systems Characterized by a Selective Expression Vector

The pETBAD, a selective expression plasmid, was used to characterize the putative TA systems in *E. coli*, as previously described [10]. First, each putative antitoxin was amplified from the genome of *S. suis* 2 using the primer pairs (0547F/R, 0790F/R, 0792F/R, 0842F/R, 0860F/R, 1035F/R, 1349F/R, 1818F/R, and 1821F/R) listed in Table S2, and the different fragments were digested, using the appropriate restriction enzymes, and cloned into the pETBAD plasmid in order to construct the pETBAD-antitoxin. Then, each putative toxin was amplified from the genome of *S. suis* 2 using the primer pairs (0548F/R, 0791F/R, 0841F/R, 0861F/R, 1034F/R, 1348F/R, 1817F/R, and 1820F/R) listed in Table S2, and they were digested using the appropriate restriction enzymes and cloned into the related, pETBAD-antitoxin, in order to construct the plasmid of the pETBAD-antitoxin-toxin (pETBAD-0547-0548, -0790-0791, -0792-0791, -0842-0841, -0860-0861, -1035-1034, -1349-1348, -1818-1817, and -1821-1820). Each putative toxin was induced by l-arabinose, and its cognate antitoxin was induced by IPTG. *E. coli* BL21 (DE3) pLysS cells, harboring the corresponding selective expression plasmids, were incubated in LB broth, supplemented with 25 μg/mL kanamycin to OD_600_ of 0.6–0.8. Each culture was serially diluted, and 5-µL drops, with concentrations ranging from 10^0^ (top) to 10^−5^ (bottom), were spotted onto four different plates, three of which were provided with various additional concentrations of l-arabinose (0.025%, 0.05%, 0.10%, and 0.20%), 1 mM IPTG, or both (induction conditions), and the fourth was provided with an additional 0.20% d-glucose (repression conditions), serving as the control.

### 4.4. Toxicity Effect of Each Toxin on E. coli Growth

Different fragments were amplified from the genome of *S. suis* 2 using the primer pairs (T0548F/R, T0791F/R, T0861F/R, T1034F/R, T1348F/R, and T1820F/R) listed in Table S2. After digesting them using the appropriate restriction enzymes, these fragments were ligated into the pBADhisA, and then the pBADhisA-toxin (pBADhisA-0548, -0791, -0861, -1034, -1348, and -1820) plasmids were constructed. *E. coli* Top10 cells, into which the related pBADhisA-toxin plasmids were transformed, were grown in LB broth, with an additional 75 μg/mL ampicillin and 0.20% d-glucose at 37 °C overnight. The next day, the cultures of the Top10 cells, carrying the plasmids of the pBADhisA-toxin or pBADhisA (control), were diluted at 1:100 in the fresh medium, supplemented with 75 μg/mL ampicillin (LB-ampicillin), and grown to OD_600_ of 0.05–0.20. Each culture was then divided into two parts. One half was grown in the presence of 0.20% d-glucose (repression conditions), while the other was grown in the presence of 0.20% l-arabinose (induction conditions). Culture growth was monitored by measuring OD_600_ every hour. On the other hand, the cultures were diluted at 1:100 in LB-ampicillin and grown to OD_600_ of 0.6–0.8. Then, they were serially diluted, and 5-µL drops, with concentrations of 10^0^ (top) to 10^−5^ (bottom), were successively spotted onto the plates with 0.20% l-arabinose or 0.20% d-glucose.

### 4.5. Effect of Antitoxin on E. coli Growth

Different fragments were amplified from the genome of SC19 using the primer pairs (0547F/R, 1349F/R, and 1821F/R) listed in Table S2. After digesting them using the appropriate restriction enzymes, these fragments were ligated into the pET30a. Then, the plasmids of the pET30a-antitoxin (pET30a-0547, -1349, and -1821) were constructed. *E. coli* BL21 (DE3) pLysS cells, into which the pBADhisA-0548 and pET30a-0547 (or pET30a), pBADhisA-1348 and pET30a-1349 (or pET30a), and pBADhisA-1820 and pET30a-1821 (or pET30a) were co-transformed, were grown in LB broth, supplemented with 75 μg/mL ampicillin and 25 μg/mL kanamycin at 37 °C overnight. The next day, the cultures of BL21 (DE3) pLysS cells, carrying the related plasmids of the pBADhisA-toxin and pET30a-antitoxin (or pET30a, as control), were diluted at 1:100 in the fresh medium, supplemented with 75 μg/mL ampicillin and 25 μg/mL kanamycin, and grown to OD_600_ of 0.2–0.3. Then, each culture was provided with an additional 1 mM IPTG and 0.20% l-arabinose, as inducers. Culture growth was monitored by measuring OD_600_ every hour. During the growth experiments, the samples of *E. coli*, harboring the related plasmids under induction or un-induction conditions, were collected in vitro at 5 h. Then, *E. coli* cells were treated using the gram stain. Microscopy images were acquired using a 100× objective, under oil-immersion.

### 4.6. Promoter Activity Assay

The primer pairs used in this experiment are listed in Table S2. The 260 bp upstream region, preceding the antitoxin (*relB*1, *relB*2, or *parD*) start codon, was selected as their promoter regions. The different fragments (antitoxin’, antitoxin-toxin’, and antitoxin-toxin) were PCR-amplified. The antitoxin’ contained the promoter region and the first 45 bp of the coding region of the antitoxin; the antitoxin-toxin’ contained the promoter region, the antitoxin coding region and the first 45 bp coding region of the toxin; and the antitoxin-toxin contained the promoter region and the full-length TA operon. Then, the ribosome-binding site (RBS) was fused to these different fragments. The PCR products were digested with *EcoR*I and *BamH*I and cloned into promoter-less *lacZ*-fusion vector pHGEI01 in order to construct the pHGEI01-antitoxin’, pHGEI01-antitoxin-toxin’, and pHGEI01-antitoxin-toxin plasmids, and the promoter-less *lacZ*-fusion vector pHGEI01 was used as the negative control (Figure S3). For the first-step PCR, we used pHGEI01-P0547-0548-F (-P1349-1348-F or -P1821-1820-F) as the forward primes, and pHGEI01-0547’-R1 (-1349’-R1 or -1821’-R1) and pHGEI01-0548’-R1 (-1348’-R1 or -1820’-R1) as the reverse primes, respectively. We then used the primer pairs, pHGEI01-P0547-0548-F (-P1349-1348-F or -P1821-1820-F)/pHGEI01-common-R2, to add the RBS (AGATCTCACACAGGAAACAGCT) sequence between the special genes (antitoxin’, antitoxin-toxin’, and antitoxin-toxin) and the *lacZ* gene for the second-step PCR. The pHGEI01-based (-*relB*1’, -*relB*1-*relE*1’, -*relB*1-*relE*1, -*relB*2’, -*relB*2-*relE*2’, -*relB*2-*relE*2, -*parD*’, -*parD*-*parE*’, and -*parD*-*parE*) plasmids were constructed, and the promoter activity assay was performed, as previously described [36]. The pHGEI01-based plasmids were transformed into the *E. coli* WM3064 strain. WM3064 cells, grown to the mid-exponential phase (OD_600_~0.6–0.7), were collected by centrifugation, washed with PBS, and resuspended in lysis buffer (0.25 M Tris/HCl, pH 7.5, 0.5% Trion-X100). Then, the soluble proteins were released by sonication, and the concentrations of the proteins were measured using a bicinchoninic acid (BCA) protein assay kit (Biosharp, Wuhan, China). The β-galactosidase activity was measured using a β-galactosidase assay kit (TIANDZ, Beijing, China), according to the manufacturer’s instructions [37].

## Figures and Tables

**Table 1:** Putative type II toxin-antitoxin (TA) systems predicted in *S. suis* SC84.

TA_No.	Toxin	Antitoxin	Strand	Distance(bp) ^1^	Domain Pair ^2^	Family
TA_1	SSUSC84_0548	SSUSC84_0547	+	−11	RHH-RelE	*relBE*/*parDE*
TA_2	SSUSC84_0791	SSUSC84_0790	−	38	-	-
TA_3	SSUSC84_0791	SSUSC84_0792	−	*−*17	RHH-RelE	*relBE*/*parDE*
TA_4	SSUSC84_0841	SSUSC84_0842	−	−1	-	SezAT
TA_5	SSUSC84_0861	SSUSC84_0860	+	14	Xre-MNT	*relBE*/*parDE*
TA_6	SSUSC84_1034	SSUSC84_1035	−	201	-	-
TA_7	SSUSC84_1348	SSUSC84_1349	−	1	RHH-RelE	*relBE*/*parDE*
TA_8	SSUSC84_1817	SSUSC84_1818	−	1	PHD-RelE	*yefM-yoeB*
TA_9	SSUSC84_1820	SSUSC84_1821	−	−11	RHH-RelE	*relBE*/*parDE*

^1^ The distance (bp) indicates the physical distance (in bp) between the putative antitoxin and toxin coding sequences, and the toxin and antitoxin genes are overlapped (−) or separated (+) by a few nucleotides; ^2^ A domain pair represents the TA protein domain pair of each antitoxin and its cognate toxin. -, it means no TA domain pair is found.

## Supplementary Materials

The following are available online at http://www.mdpi.com/2072-6651/10/11/467/s1: Figure S1: Genomic location of nine putative type II toxin-antitoxin (TA) systems in the chromosome of *S. suis* SC84. Figure S2: Genetic organization of nine putative type II toxin-antitoxin (TA) systems. Figure S3: Schematic representation of the constructed reporter systems for the promoter activity assay. Table S1: Bacterial strains and plasmids used in this study. Table S2: Primers used in this study.
