# Supplementary material for: Identification of Three Type II Toxin-Antitoxin Systems in Streptococcus suis Serotype 2
Source: Toxins (Basel). 2018 Nov 13;10(11):467. doi: 10.3390/toxins10110467 (PMC6266264; doi:10.3390/toxins10110467)
Supplement: Supplementary file 1 [file toxins-10-00467-s001.zip › toxins-384791-SI.pdf]

Supplementary Materials: Identification of novel three Type II Toxin-Antitoxin Sysems in *Stretococcus suis* serotype 2

Jiali Xu, Nian Zhang, Manman Cao, Sujing Ren, Ting Zeng, Minglu Qin, Xigong Zhao, Fangyan Yuan, Huanchun Chen and Weicheng Bei

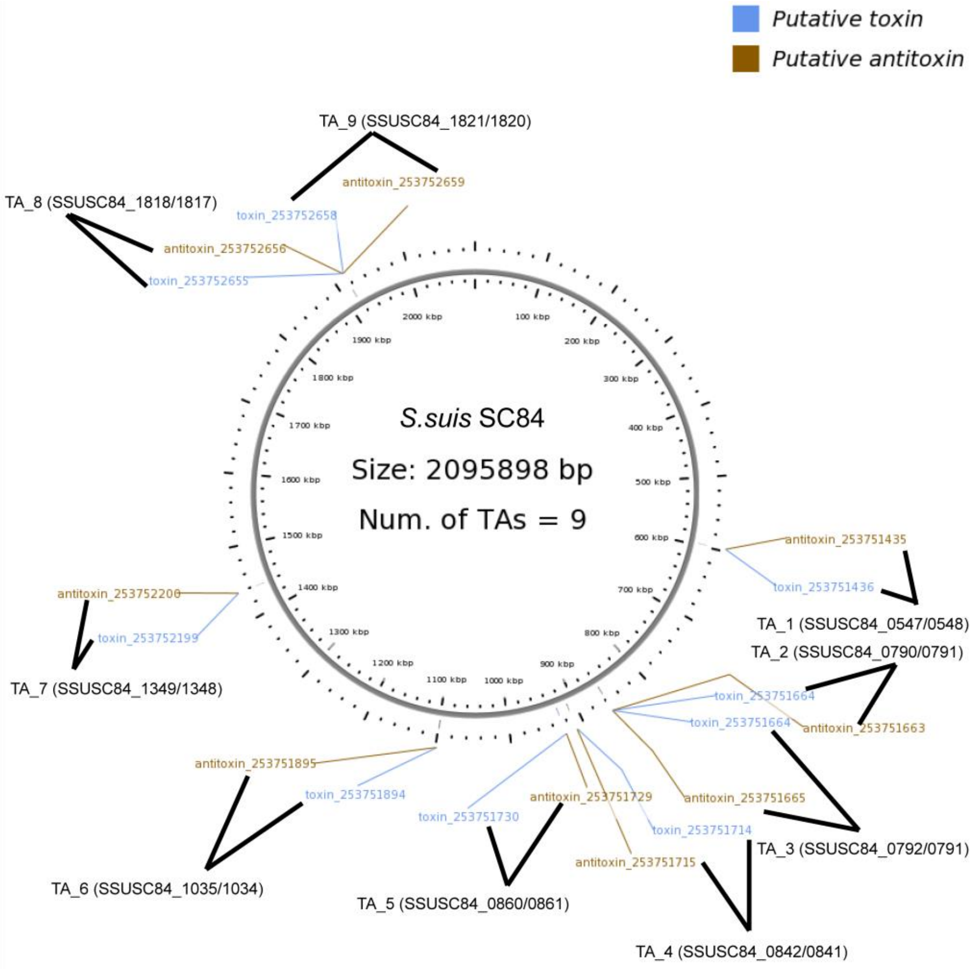

Figure S1. Genomic location of nine putative type II toxin-antitoxin (TA) systems in the chromosome of *S. suis* SC84. The ID numbers of each putative toxin or antitoxin are included (toxin_No. or antitoxin_No.) in the figure.

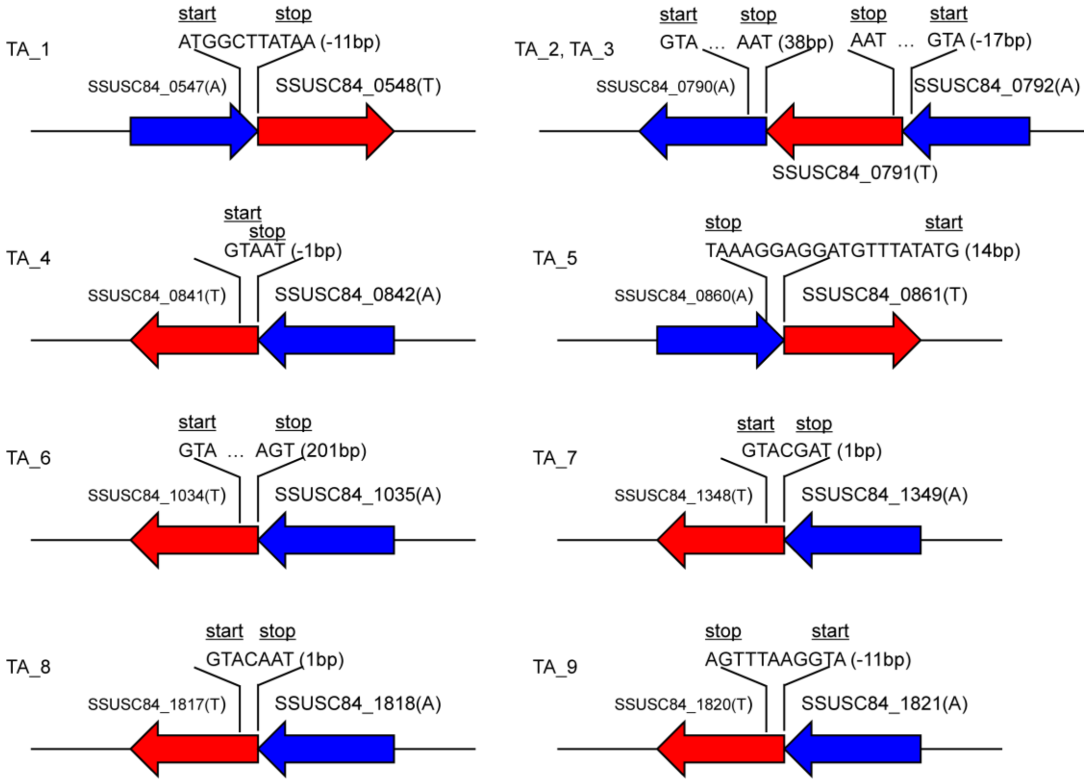

Figure S2. Genetic organization of nine putative type II toxin-antitoxin (TA) systems. The arrows indicate the direction of the transcription and do not represent the exact length. Red arrows represent putative toxins, and blue arrows represent putative antitoxins.

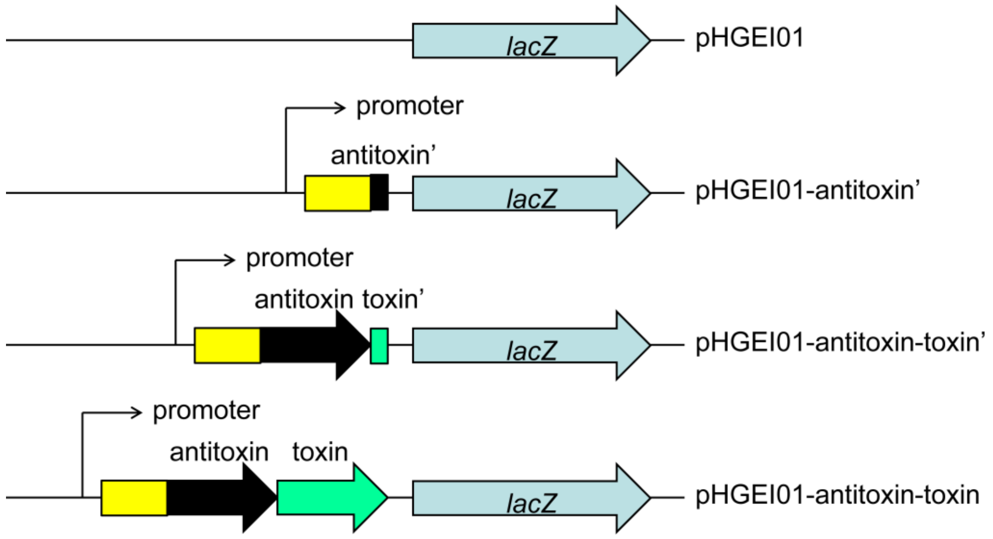

Figure S3. Schematic representation of the constructed reporter systems for the promoter activity assay.

Table S1. Bacterial strains and plasmids used in this study.

Strain or plasmid	Relevant characteristics [a]	Source or reference
**Strains**		
SC19	Virulent strain isolated from the brain of a dead pig; Serotype 2	Laboratory collection
E. coli		
DH5α	Cloning host for recombinant vector	TransGen
TOP10	The expression host for pBADhisA and its derivative	TransGen
BL21 (DE3) pLysS	The expression host for pET-30a, pETBAD and their derivative	TransGen
WM3064	*thrB*1004 *pro thi rpsL hsdS lacZ*ΔM15 RP4-1360 Δ(*araBAD*)567 Δ*dapA*1341::[*erm pir*(wt)]	Metcalf, W.; UIUC
**Plasmids**		
pETBAD	Selective expression vector with the pET-30a background; Kan^R	[10]
pETBAD-0547-0548	pETBAD containing the 0547 and 0548 genes	This study
pETBAD-0790-0791	pETBAD containing the 0790 and 0791 genes	This study
pETBAD-0792-0791	pETBAD containing the 0792 and 0791 genes	This study
pETBAD-0842-0841	pETBAD containing the 0842 and 0841 genes	This study
pETBAD-0860-0861	pETBAD containing the 0860 and 1820 genes	This study
pETBAD-1035-1034	pETBAD containing the 1035 and 1034 genes	This study
pETBAD-1349-1348	pETBAD containing the 1349 and 1348 genes	This study
pETBAD-1818-1817	pETBAD containing the $yefM_{Ssu}$ and *yoeB* genes	This study
pETBAD-1821-1820	pETBAD containing the 1821 and 1820 genes	This study
pBADhisA	Expression vector; Amp^R	Invitrogen
pBADhisA-0548	pBADhisA containing the SSUSC84_0548 gene	This study
pBADhisA-0861	pBADhisA containing the SSUSC84_0861 gene	This study
pBADhisA-1348	pBADhisA containing the SSUSC84_1348 gene	This study
pBADhisA-1820	pBADhisA containing the SSUSC84_1820 gene	This study
pET-30a	Expression vector; Kan^R	Novagen
pET-30a-0547	pET-30a containing the SSUSC84_0547 gene	This study
pET-30a-0860	pET-30a containing the SSUSC84_0860 gene	This study
pET-30a-1349	pET-30a containing the SSUSC84_1349 gene	This study
pET-30a-1821	pET-30a containing the SSUSC84_1821 gene	This study
pHGEI01	Kan^R, R6K *ori*, pHGC01 containing the full-length *E. coli lacZ* gene	[28]
pHGEI01-*relB*1′	pHGEI01 containing the promoter of SSUSC84_0547/0548	This study
pHGEI01-*relB*1-*relE*1′	pHGEI01 containing the promoter of SSUSC84_0547/0548 and SSUSC84_0547	This study
pHGEI01-*relB*1-*relE*1	pHGEI01 containing SSUSC84_0547/0548 and its promoter	This study
pHGEI01-*relB*2′	pHGEI01 containing the promoter of SSUSC84_1349/1348	This study
pHGEI01-*relB*2-*relE*2′	pHGEI01 containing the promoter of SSUSC84_1349/1348 and SSUSC84_1349	This study
pHGEI01-*relB*2-*relE*2	pHGEI01 containing SSUSC84_1349/1348 and its promoter	This study
pHGEI01-pa*rD*′	pHGEI01 containing the promoter of SSUSC84_1821/1820	This study
pHGEI01-pa*rD*-*parE*′	pHGEI01 containing the promoter of SSUSC84_1821/1820 and SSUSC84_1821	This study
pHGEI01-pa*rD*-*parE*	pHGEI01 containing SSUSC84_1821/1820 and its promoter	This study

[a] Amp^R and Kan^R indicate ampicillin and kanamycin resistance, respectively.

Table S2. Primers used in this study.

Primer	Sequence (5′–3′) [a]	Target gene
0547F	CGGCGGTACCATGGGAATTGTATCCTTGCG	SSUSC84_0547
0547R	CGCCGAATTCTTATAAGCCATCTTCTAACTCCG	
0548F	CCCCGTCGACATGGCTTATAAGCTTGTTCTCAG	SSUSC84_0548
0548R	CGCCGAATTCTCATTTTTTATAGATTTCTTTCCG	
0790F	CGGCGGTACCATGAAAAAAGAGAAGATTTATATTGTT	SSUSC84_0790
0790R	CCGCGAGCTCTCATTTGATCAAGTACCCCAC	
0791F	CCCCAAGCTTATGAAGACCTGTTATAAACTCGTAC	SSUSC84_0791
0791R	CCGCGAGCTCTTATATACTTCCATTTCTCATTTTTAT	
0792F	CGGCGGTACCATGGCAGCTATTACATTGAAAGT	SSUSC84_0792
0792R	CCGCGAGCTCTTATAACAGGTCTTCATCCGTCA	
0841F	CCCCAAGCTTATGAGGTTGGAAGAATTTAGTGA	SSUSC84_0841
0841R	CCGCGAGCTCTCATTTCTTCTCTCCATTAGCTG	
0842F	CGGCGGTACCATGATCGGAGACAATATAAAATCA	SSUSC84_0842
0842R	CGCCGAATTCTTAAGCCACCACCCGATG	
0860F	CGGCGGTACCATGACTACGGCAGAAATGATTA	SSUSC84_0860
0860R	CGCCGAGCTCTTATTCGTTAGATATTTTTATTTCTTCT	
0861F	CCCCGTCGACATGGTAGGTAATGTTATTCAAATAGTA	SSUSC84_0861
0861R	CCGCGAGCTCTCATATTTGTTTTCTTTCATCTGA	
1034F	CCCCAAGCTTATGCAGATTGATCCTATACGTTT	SSUSC84_1034
1034R	CCGCGAGCTCTTATCCTTTCTGGTGCAAAGG	
1035F	CGGCGGTACCATGTCAAACATTGAACAACAGTT	SSUSC84_1035
1035R	CGCCGAATTCTCAAAACTGCTCCGAGAATC	
1348F	CCCCGTCGACATGTATCATTTAGAGTATTCTAAAAAGG	SSUSC84_1348
1348R	CGCCGAATTCTCAATAGACATCTTTACGATGTCC	
1349F	CGGCGGTACCATGAGTACAGTGACAATCAGATTAAAT	SSUSC84_1349
1349R	CGCCGAATTCCTATAATCCTAGCTCTTTCTTAAAATC	
1817F	CCCCAAGCTTATGGGAATCCATTTTACAGAC	SSUSC84_1817
1817R	CCGCGAGCTCTCACCTATAATGATCCTTTAGCG	
1818F	CGGCGGTACCATGGAAGCTATTGTATATTCCCAT	SSUSC84_1818
1818R	CGCCGAATTCTTAGTCCGCCTCTATCAGGTT	
1820F	CCCCAAGCTTATGGAATTTGAGTCTTATTCAGTG	SSUSC84_1820
1820R	CCGCGAGCTCTCACTTCAACAACTTCACATAATC	
1821F	CGGCGGTACCTTGATAACTATAAATAAAGATGCCC	SSUSC84_1821
1821R	CGCCGAATTCTCAAATTCCATACTTCTCTTTGAC	
T0548F	CCGCGAGCTCATGGCTTATAAGCTTGTTCTCAG	SSUSC84_0547
T0548R	CGCCGAATTCTCATTTTTTATAGATTTCTTTCCG	
T0791F	CCGCGAGCTCATGAAGACCTGTTATAAACTCGTAC	SSUSC84_0791
T0791R	CGCCGAATTCTTATATACTTCCATTTCTCATTTTTAT	
T0861F	CCGCGAGCTCATGGTAGGTAATGTTATTCAAATAGTA	SSUSC84_0861
T0861R	AAAACTGCAGTCATATTTGTTTTCTTTCATCTGA	
T1034F	CCGCGAGCTCATGCAGATTGATCCTATACGTTT	SSUSC84_1034
T1034R	CGCCGAATTCTTATCCTTTCTGGTGCAAAGG	
T1348F	CCGCGAGCTCATGTATCATTTAGAGTATTCTAAAAAGG	SSUSC84_1348
T1348R	CGCCGAATTCTCAATAGACATCTTTACGATGTCC	
T1820F	CCGCGAGCTCATGGAATTTGAGTCTTATTCAGTG	SSUSC84_1820
T1820R	CGCCGAATTCTCACTTCAACAACTTCACATAATC	
pHGEI01-P0547-0548-F	CCGGAATTCAGTTTTTCCTTTATTACGTCGAC	
pHGEI01-0547′-R1	AGCTGTTTCCTGTGTGAGATCTAATTTCTTCTTCTGCATCGTTT	
pHGEI01-0548′-R1	AGCTGTTTCCTGTGTGAGATCTCAACTGCTTCAAAGCATCATC	
pHGEI01-0547-0548-R1	AGCTGTTTCCTGTGTGAGATCTTCATTTTTTATAGATTTCTTTCCG	
pHGEI01-common-R2	CGCGGATCCGTAATCATGGTCATAGCTGTTTCCTGTGTGAGATCT	
pHGEI01-P1349-1348-F	CCGGAATTCAGTAAGAACAAGTCTGGAAATTTC	
pHGEI01-1349′-R1	AGCTGTTTCCTGTGTGAGATCTGAATACTTCTTCTTCTTGATTTAATCT	
pHGEI01-1348′-R1	AGCTGTTTCCTGTGTGAGATCTCATAATTTGCTTTTGAGCCTT	
pHGEI01-1349-1348-R1	AGCTGTTTCCTGTGTGAGATCTTCAATAGACATCTTTACGATGTCC	
pHGEI01-P1821-1820-F	CCGGAATTCGCAGAGCCGTTTGTTTCATAT	
pHGEI01-1821′-R1	AGCTGTTTCCTGTGTGAGATCTATCAGTTTTAAAATTTACTTGGGC	
pHGEI01-1820′-R1	AGCTGTTTCCTGTGTGAGATCTCTCCACTGCTGGAGCAAGTAT	
pHGEI01-1821-1820-R1	AGCTGTTTCCTGTGTGAGATCTTCACTTCAACAACTTCACATAATCA	

[a] The underlined sequences are restriction sites.
